# 65 Reducing High Mobility Group Box 1 Activities to Prevent Scald Burn Wound Progression in Rats

**DOI:** 10.1093/jbcr/iraf019.065

**Published:** 2025-04-01

**Authors:** Sophia Lee, Amina El Ayadi, Steven Wolf, Nisha Garg, Juquan Song

**Affiliations:** University of Texas Medical Branch, John Sealy School of Medicine; University of Texas Medical Branch; University of Texas Medical Branch; University of Texas Medical Branch; University of Texas Medical Branch

## Abstract

**Introduction:**

Severe burns trigger hyperinflammatory and hypermetabolic responses, leading to systemic organ damage. High mobility group box 1 (HMGB1) belongs to damage associated molecular pattern (DAMPs) which are released from burn wound sites and contribute to the innate immune system activation. We previously observed that neutralizing the circulating HMGB1 mitigated muscle loss in a severely burned rats, and we hypothesize that the systemic protective effect reflects on the wound sites as well. This study investigated wound healing in scald burn rats treated with an anti-HMGB1 antibody.

**Methods:**

Male Sprague Dawley rats were divided into three groups: sham burn (n=5), burn with vehicle treatment (n=8), and burn with anti-HMGB1 treatment (n=8). Rats in the burn and treatment groups received 30% total body surface area burns and were treated with either chicken IgY (vehicle group) or anti-HMGB1 antibody. Skin samples were collected at 3 and 14 days post-burn for histological process. The histological morphology including wound depth (epithelial, dermal, and panniculus carnosus muscle thickness) and size were measured via light microscopy and ImageJ analysis. The burn wound edge and center were also compared to assess the burn wound progression in each sample.

**Results:**

The epithelial thickness was significantly greater in the anti-HMGB1 group by day 14 compared to the control (58µm ± 22µm vs. 21µm ± 3µm; p< 0.01). Dermal thickness was also significantly increased in the anti-HMGB1 group compared to the vehicle group on day 14 (1.7mm ± 0.23mm, vs. 1.4mm ± 0.25mm; p< 0.05), a marker of the wound’s proliferative phase. Moreover, dermal thickness decreased in the vehicle group compared to the control on days 3 (1.5mm ± 0.36mm vs. 2.0mm ± 0.21mm; p< 0.05) and 14 (1.4mm ± 0.25mm vs. 2.0mm ± 0.21mm; p< 0.01). In comparing the burn wound center and edge, the muscle thickness difference of the panniculus carnosus was less in the anti-HMGB1 group compared to the vehicle (-6.4% ± 1.5% vs. -70.9% ± 25%; p< 0.05), with significant preservation of this key muscle layer needed for collagen production and healing. Finally, wound size reduction was significantly greater in the anti-HMGB1 group compared to the vehicle by day 3 (-6.1% ± 1.3% vs. -4.8% ± 0.95%; p< 0.05), indicating enhanced wound contraction.

**Conclusions:**

Anti-HMGB1 treatment improves burn wound healing by increasing epithelial and dermal thickness, reducing muscle loss, and decreasing wound size.

**Applicability of Research to Practice:**

These results suggest a critical role for HMGB1 in burn injuries and potential for targeted therapies in severe burn management. Future directions include examining HMGB1 expression and vasculature to further elucidate HMGB1’s role in wound healing in burns.

**Funding for the Study:**

This work was supported by the Remember 15 research endowment at Surgery department, the Clinical and Translational Science Award (UL1 TR001439) from the National Center for Advancing Translational Sciences at the National Institutes of Health (NIH), and the Trauma Research and Combat Casualty Care Collaborative (TRC4 #CON32808).

